# Stable Field Emissions from Zirconium Carbide Nanoneedle Electron Source

**DOI:** 10.3390/nano15020093

**Published:** 2025-01-09

**Authors:** Yimeng Wu, Jie Tang, Shuai Tang, You-Hu Chen, Ta-Wei Chiu, Masaki Takeguchi, Ayako Hashimoto, Lu-Chang Qin

**Affiliations:** 1 Research Center for Energy and Environmental Materials, National Institute for Materials Science, Tsukuba 305-0047, Ibaraki, Japan; wu.yimeng@nims.go.jp (Y.W.); chenyouhu@hotmail.com (Y.-H.C.); chiu.tawei@nims.go.jp (T.-W.C.); takeguchi.masaki@nims.go.jp (M.T.); hashimoto.ayako@nims.go.jp (A.H.); 2 Graduate School of Science and Technology, University of Tsukuba, Tsukuba 305-8577, Ibaraki, Japan; 3 State Key Laboratory of Optoelectronic Materials and Technologies, Guangdong Province Key Laboratory of Display Material and Technology, School of Electronics and Information Technology, Sun Yat-sen University, Guangzhou 510275, China; tangsh58@mail.sysu.edu.cn; 4 Department of Physics and Astronomy, University of North Carolina at Chapel Hill, Chapel Hill, NC 27599-3255, USA

**Keywords:** zirconium carbide, nanoneedle, electron source, stable field emission

## Abstract

In this study, a single zirconium carbide (ZrC) nanoneedle structure oriented in the <100> direction was fabricated by a dual-beam focused ion beam (FIB-SEM) system, and its field emission characteristics and emission current stability were evaluated. Benefiting from controlled fabrication with real-time observation, the ZrC nanoneedle has a smooth surface and a tip with a radius of curvature smaller than 20 nm and a length greater than 2 μm. Due to its low work function and well-controlled morphology, the ZrC nanoneedle emitter, positioned in a high-vacuum chamber, was able to generate a single and collimated electron beam with a current of 1.2 nA at a turn-on voltage of 210 V, and the current increased to 100 nA when the applied voltage reached 325 V. After the treatment of the nanoneedle tip, the field emission exhibited a stable emission for 150 min with a fluctuation of 1.4% and an emission current density as high as 1.4 × 10^10^ A m^−2^. This work presents an efficient and controllable method for fabricating nanostructures, and this method is applicable to the transition metal compound ZrC as a field emission emitter, demonstrating its potential as an electron source for electron-beam devices.

## 1. Introduction

Cold field emissions, also known as room-temperature field emissions, refer to the process of electron emissions from a cold cathode under the influence of a strong electric field. This quantum phenomenon has been the focus of extensive scientific investigations over the past ninety years [1,2,3,4]. As early as the late 19th century, it was first noted that strong electric fields could induce electron emissions from surfaces and that the current between two metals in a vacuum exceeds theoretical predictions [5]. Fowler and Nordheim later developed the quantum mechanical theory to describe the field emission process in bulk metals, and a set of equations, commonly referred to as the Fowler–Nordheim (F-N) equations, have been frequently used as a good approximation to describe field emissions from crystalline materials like metals and semiconductors [6]. As the research progressed, field emission electron sources, initially employed in rudimentary applications such as radar and microwave amplifiers [7,8,9], have evolved to meet the demands of applications such as electron microscopy, electron beam lithography, X-ray tubes, and displays [10,11,12,13]. These developments have been applied to meet the growing demands of miniaturized and highly efficient electron sources in modern technologies.

In contrast to thermionic emissions, where filaments are heated to high temperatures (1000–3000 °C) to supply electrons with sufficient energy to overcome the material’s work function [14], cold field emissions apply a high electric field to the surface of the material, effectively narrowing the potential barrier to a few nanometers. This allows free electrons at the Fermi level (following the Dirac–Einstein distribution) to tunnel through the energy barrier via quantum tunneling and be emitted into the vacuum state at room temperature [5,15,16,17,18]. This mechanism provides field emissions with several advantages: a high response speed, narrow energy spread, and low power consumption. To achieve the extremely high local electric field required for inducing field emission, often reaching a few V/nm, reducing the diameter of the emitter tip can significantly increase the electron flux density near the tip [19], effectively reducing the macroscopic extraction voltage by an order of magnitude from several kilovolts [20,21]. On the other hand, cathode materials with low work functions exhibit lower energy spread, resulting in electrons that are more conducive to tunneling under smaller electrostatic forces [22,23]. The ongoing development of electron emitters with a low work function and structures with a high aspect ratio has been a key focus in the development of field emission electron sources.

In recent decades, significant efforts have been directed toward the systematic investigation of materials with a low work function and high melting point using the one-dimensional nanostructure for application as field emission electron sources. Due to their specific geometry and controllable composition and structure, one-dimensional nanostructures have been considered promising for field emission electron sources [24,25,26,27]. One-dimensional boride, nitride, and carbide nanostructures have been extensively studied as stable cold field emission electron sources [28,29,30,31,32]. Zhao et al. reviewed the synthesis methods of Si_3_N_4_ nanowires and their applications and prospects in optoelectronics [32]. Tang et al. successfully produced HfC nanowire field emitters with long-term stable emissions, and their further research and development led to the realization of long-term stable emissions from CeB_6_ and LaB_6_ nanoneedle field emitters [29,30,31]. Zirconium carbide (ZrC) is a well-known refractory ceramic material with a NaCl-type crystal structure and has been highly valued for its potential in field emission applications due to its low work function (3.6 eV), high melting temperature (3532 °C), and excellent chemical stability [33,34,35,36]. Makie et al. demonstrated the field emission capability of ZrC single crystals, which were obtained via arc floating zone refinement from sintered stock, showing that ZrC emitters could be operated under pressures far exceeding those typically used for field emission cathodes as early as the late 1980s [37]. Recently, our team has successfully utilized a single ZrC nanowire as a field emission electron source, which was synthesized via chemical vapor deposition (CVD) and assembled into an electron emitter using a nano-manipulation system under an optical microscope [38], demonstrating the great potential of the ZrC nanowire as a field emission filament. However, nanowire structures must face significant challenges in industrial applications. The complexities of the synthesis and assembly processes considerably impact the manufacturing efficiency of nanowire emitters. Additionally, the points of contact with the platform could result in inadequate structural robustness. The vibrations of the emitter tip during emission also hinder their ability to provide a stable emission current under practical operating conditions.

In this study, we report the successful fabrication and development of a single ZrC nanoneedle as a field emission point electron source and detailed characterizations of its emission properties and emission current stability. The ZrC nanoneedle emitter showed an extraction voltage of 210 V and achieved an emission current density as high as 1.4 × 10^10^ A m^−2^, which can be attributed to its optimized morphology and surface structure with a low work function.

## 2. Experimental Section

**Figure 1:**
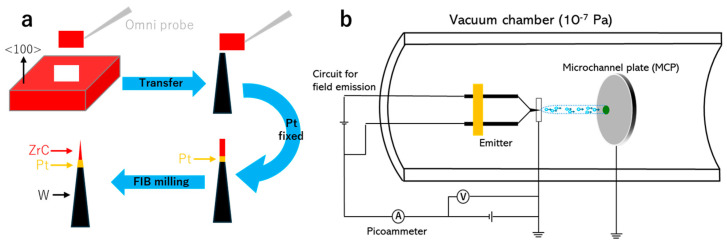
Schematic of (**a**) the fabrication process of ZrC nanoneedles using the FIB-SEM system. (**b**) The experimental setup for the field emission test.

Following the successful stabilization of the field emission current from ZrC nanowire emitters, we developed a two-step fabrication process employing ion milling to produce ZrC emitters with improved structural robustness [30,31]. Figure 1a presents a schematic of our controlled fabrication method for producing ZrC nanoneedle field emission electron sources utilizing a dual-beam focused ion beam (FIB-SEM, Helios 650, FEI, Hillsboro, OR, USA) system.

In the first step, a (100)-oriented ZrC bulk crystal was sectioned into a lamella with a dimension of 3 × 10 μm and transferred using an Omni probe. It should be noted that the rocking curve measurement of the ZrC crystal was performed on the ZrC (200) peak (2θ = 38.44°). In this context, omega (ω) refers to the rotation angle of the sample relative to the incident X-ray beam, and the theoretical omega was calculated as half of 2θ with θ being the Bragg angle and satisfying Bragg’s law. The experimental omega, obtained from the rocking curve scan, indicated an “off-angle along the incident beam direction”, which was estimated to be less than ±0.06°, suggesting the near-perfect alignment of the sample along the vertical axis. The lamella was subsequently mounted onto a tungsten (W) needle tip, which has a pre-prepared planar platform created by FIB milling. Platinum (Pt) was deposited at the contact point between the ZrC lamella and the W needle using electron beam-induced deposition, ensuring the stability and reliability of the ZrC emitter. Finally, Ga-ion milling was employed to sharpen the ZrC lamella into a nanoneedle as an emitter. The crystallographic orientation of the ZrC crystal was measured by X-ray diffraction (XRD; SmartLab, Rigaku, Tokyo, Japan). Scanning electron microscopy (SEM; JSM-6500F, JEOL, Tokyo, Japan) and transmission electron microscopy (TEM; JEM-ARM200F, JEOL, Tokyo, Japan) were used to characterize the microstructure of the ZrC nanoneedle. This fabrication method for electron sources offers the advantages of high efficiency and controllability, significantly reducing the preparation time to 50 min for a single filament. Additionally, progress can be monitored in real-time and regulated by adjusting the milling current.

The field emission characteristics of the ZrC nanoneedle were evaluated in a high-vacuum chamber (1 × 10^−7^ Pa), as shown in Figure 1b, designed for both field emissions and thermal flashing procedures. Prior to measurement, thermal flashing pretreatment was applied to remove the adsorbates and contaminants on the ZrC nanoneedle surface. A negative field was applied to the ZrC nanoneedle emitter to extract electron emissions and record different current values at the picometer. A grounded microchannel plate (MCP) was placed 5 cm from the emitter to record the emission current and observe the field emission pattern.

## 3. Results and Discussion

**Figure 2:**
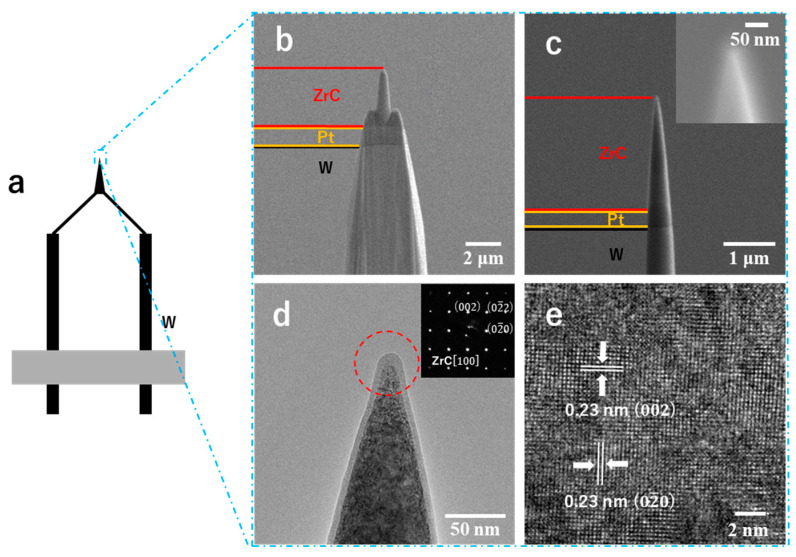
(**a**) Schematic of the ZrC nanoneedle field emission electron source with hairpin structure. (**b**) SEM image of ZrC nanoneedle during the process of Ga-ion milling. (**c**) SEM image of ZrC nanoneedle after fabrication was completed. (**d**) TEM image and electron diffraction pattern (inset) of the sharpened ZrC nanoneedle tip. (**e**) High-resolution TEM image near the surface region.

A field emission electron source composed of a sharpened tip supported with a hairpin filament is illustrated in Figure 2a. Tungsten is typically used as the cathode material of electron sources, and the cathode filament begins emitting electrons when an electric field is created. The anode, maintained at a positive potential relative to the filament, generates a strong electric field that attracts and accelerates the electrons toward it. Some electrons pass through the anode and continue traveling to the column toward the detector. In this study, we replaced the W tip with ZrC to act as the electron emitter. Figure 2b,c present the SEM images of the emitter tip during and after the fabrication process, respectively. The images reveal that Ga-ion milling effectively sharpened the emitter tip. The conical shape of the electron emitter tip, in combination with the hairpin structure, ensured the structural robustness of the single nanoneedle without severe vibration affecting the field emissions. In both images, three regions of distinct contrast correspond to the ZrC tip, Pt deposition, and W needle. These regions are highlighted and distinguished using different colors for clarity. The fabricated nanoneedle possesses a total length exceeding 10 µm with a tip of a radius of curvature less than 20 nm. Following real-time observations using a controlled milling current, the high-magnification SEM image of the nanoneedle tip exhibited a sharpened tip apex with a radius of curvature of approximately 20 nm and a smooth surface, as shown in the inset of Figure 2c. The morphology and curvature discussed above were also confirmed in the TEM image of the emitter’s tip, as shown in Figure 2d. Additionally, due to the extended exposure of the nanoneedle sample to the air during transfer to the TEM grid and the impact of Ga-ion milling on the surface, an oxidized amorphous layer with a thickness of approximately 5 nm was observed on the tip surface. The formation of the oxidized amorphous layer was initially due to the amorphous layer generated by Ga ion bombardment during the fabrication process, followed by the oxidation layer formed during surface exposure to oxygen in the transfer process. Typically, this oxidized amorphous layer can be removed via thermal flashing and high-current field emissions during surface pretreatment. The inset in Figure 2d shows an electron diffraction pattern from the tip region, corresponding to the <100> zone axis of the ZrC crystal. The uppermost part of the ZrC nanoneedle retained the single crystallinity of the <100> oriented single crystal. In addition, the high-resolution TEM image near its surface region (Figure 2e) shows a crystal lattice spacing of 0.23 nm in two directions, corresponding to the {200} lattice spacing of the ZrC crystal.

**Figure 3:**
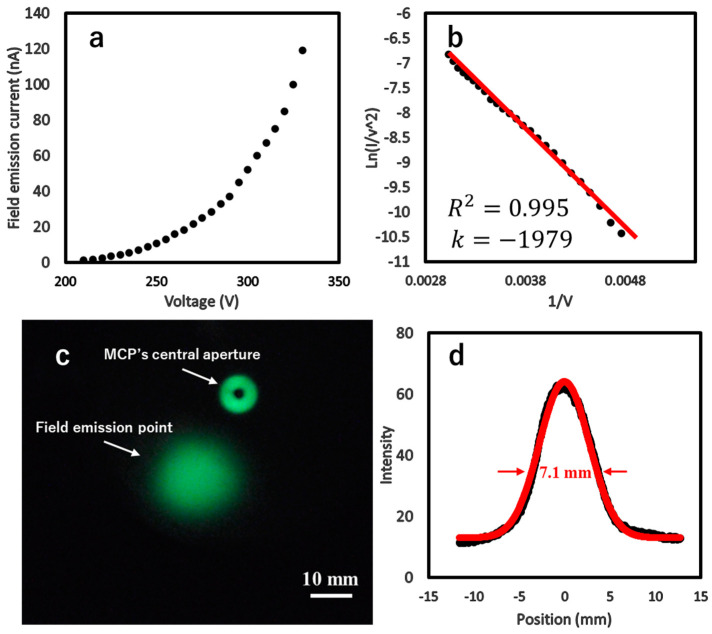
Field emission characteristics of the ZrC nanoneedle emitter. (**a**) I-V curve of field emissions and (**b**) its corresponding F-N plot. (**c**) FEM pattern of the ZrC nanoneedle with a single emission spot in the axial direction. (**d**) Field emission intensity following a Gaussian distribution with FWHM of 7.1 mm.

The prepared ZrC nanoneedle emitter was placed in a high-vacuum chamber (1 × 10^−7^ Pa), where a gradually increasing electric field was applied to collect the emission current from the emitter tip for the evaluation of its field emission properties. Figure 3a shows the relationship between the field emission current (I) and extraction voltage (V). The ZrC nanoneedle emitter exhibited a field emission current of 1.2 nA at a turn-on voltage of 210 V, and the current increased to 100 nA as the applied voltage reached 325 V. The field emission characteristics were analyzed by the Fowler–Nordheim (F-N) equation [5].

(1) $$I=\frac{Ac_{1}F^{2}}{\phi }\exp\left(\frac{-c_{2}\phi^{\frac{3}{2}}}{F}\right)$$

Here, I is the field emission current, A is the emission area, ϕ is the work function of the emitter surface, $c_{1}=1.54\times 10^{6}\;$AeV V^−2^ and $c_{2}=6.83\times 10^{9}$ eV^−3/2^ V m^−1^ are constants, and F is the electric field applied on emitter tip, which can be expressed as follows:

(2) F=βV

Here, β is the field enhancement factor, which is a proportionality factor between the extraction voltage V and the electric field F, and is determined by the local geometry of the electron emitter. The linearized relationship (F-N plot) between $\ln\left(I/V^{2}\right)$ and 1/V is calculated as follows:

(3) $$\ln\left(\frac{I}{V^{2}}\right)=\frac{S}{V}+b$$

This produced the following slope:

(4) $$S=-6.83\times 10^{9}\phi^{3/2}/\beta$$

The F-N plot shown in Figure 3b demonstrates excellent linearity with an R^2^ coefficient of 0.995, indicating that the field emission behavior of the ZrC nanoneedle emitter closely follows the traditional cold field emission model described by the F-N equation. Furthermore, by substituting the slope S = −1979 V and the work function of ZrC(100) ϕ=3.6 eV into Equation (4), we calculated the local field enhancement factor *β* = 2.18 × 10^7^ m^−1^. This allowed us to evaluate the emitter tip’s electric field at each extraction voltage during the field emission. The ZrC nanoneedle emitter, produced by using our efficient and controllable fabrication process, exhibited a field enhancement factor *β* that was an order of magnitude higher than the W(310) filament with *β* = 1 × 10^6^ m^−1^.

The emitter gave off a single electron beam during testing, which can be observed in the field emission microscope (FEM) pattern shown in Figure 3c. The center of the image clearly shows a single emission point on the microchannel plate (MCP). Located at the upper right of this emission point is the MCP’s central aperture, which is positioned to collect probe current and exhibit fluorescence due to its positive potential. The intensity of the field emission beam in the FEM pattern follows a Gaussian distribution (Figure 3d), exhibiting a diameter of 7.1 mm at its full width at half maximum (FWHM) and a corresponding semi-angle of divergence of 71 mrad. The Gaussian distribution curve (red) is fitted to the intensity profile of the FEM pattern (black) for evaluations. Based on the parameters of the FEM, we determined that the electrons were emitted from a region of approximately 3.7 nm^2^ at the ZrC nanoneedle tip. This accounts for the fact that when the field emission current reached 50 nA at 300 V, a local electric field of 6.5 V nm^−1^ at the nanoneedle tip was created. The reduced brightness of the ZrC emitter was calculated using the following formula [39,40]:

(5) $$B_{r}=\frac{1.44J}{\pi d}$$

where J is the areal density of the emission current and the variable is given as follows:

(6) $$d=9.76\times 10^{-11}\frac{F}{\phi^{1/2}t(y)}$$

This equation represents the transverse energy, while the function t(y), related to the image potential in F-N theory, can be approximated as $t\left(y\right)=1+0.1107y^{1.33}.\;y=3.79\times 10^{-5}F^{0.5}\phi^{-1}$. By substituting a work function of 3.6 eV, a local electric field of 6.5 V nm^−1^, and an emission area of 3.7 nm² into the above equations, we obtained the transverse energy d=0.39 eV. When the field emission current reached 50 nA at 300 V, the current density was $J=1.4\times 10^{10}$ A m^2^, and the reduced brightness of this nanoneedle emission current was $1.4\times 10^{10}$ A m^2^ sr^−1^ V^−1^. The significantly higher brightness and current density of the ZrC nanoneedle emitter were actually obtained in a relatively lower vacuum (10^−7^ Pa) compared to the commercial W(310) filament. Such performance highlights the ZrC nanoneedle’s potential as an effective field emission source, particularly in environments where W filaments struggle to maintain stable emissions.

Benefiting from its optimized morphology and low work function of the tip material, the ZrC nanoneedle emitter showed an extraction voltage of 210 V, a field enhancement factor β of 2.18 × 10^7^, and an emission current density as high as 1.4 × 10^10^ A m^−2^. Compared to recently developed InSb nanowire arrays and WS_2_ nanotube arrays [41,42], both arrays had turn-on voltages in the range of tens of volts due to the short distance from the anode (500 nm). The ZrC nanoneedle emitter not only benefits from the exceptionally high field enhancement inherent to single-point field emissions but also achieves a current density that is more than an order of magnitude higher, ensuring a high brightness for cold field emission sources. When compared with conventional W and LaB_6_ single emitters, the ZrC emitter significantly outperforms the W needle, which has a turn-on voltage exceeding 2000 V and a field enhancement factor β on the order of 1 × 10^6^. Although LaB_6_ nanoneedles exhibit comparable current density and a lower local electric field, their lower work function enables field emissions at an even lower voltage of 165 V. The stability under high currents has always been a challenge that LaB_6_ needs to address [30,43].

After confirming the practicality of the ZrC nanoneedle emitter’s field emission characteristics, the stability of the field emission current becomes another crucial parameter to consider for practical use in microelectronic devices. The most commonly used commercial field emitter, the W(310) filament, requires operation in an extremely high vacuum (EHV, 10^−9^ Pa) due to its instabilities and significant decay in field emission currents. This stringent requirement of the vacuum level substantially increases both the cost and efforts of maintenance of the devices [44,45].

**Figure 4:**
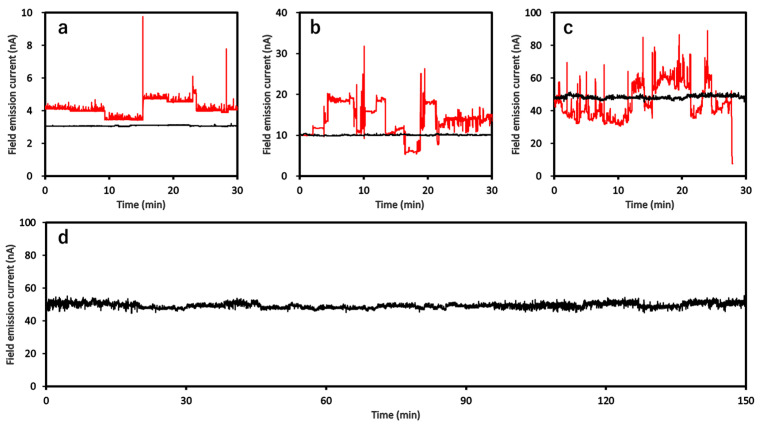
The 30 min field emission stability before (red line) and after (black line) the ZrC nanoneedle emitter stabilized under emission currents of (**a**) 3 nA, (**b**) 10 nA, and (**c**) 50 nA with fluctuations of 0.30%, 0.31%, and 0.60%, respectively. (**d**) Long-term stability with a fluctuation of 1.41% after 2.5 h of measurement.

Figure 4a–c display three comparisons of before (red line) and after (black line) stabilizing the ZrC nanoneedle emitter under field emission currents of 3 nA, 10 nA, and 50 nA at a vacuum of 1 × 10^−7^ Pa, respectively. During this process, thermal flashing and high current treatment were applied to clean the adsorbates and contaminants on the emitter’s tip surface, effectively stabilizing its surface structure. As a result, the initially stepped and fluctuating field emission current (red line) gradually diminished, eventually showing a smooth and steady current (black line). The current stabilities, calculated by the formula $\sum \left[{\left(I_{i}-\bar{I}\right)}^{2}\right]/[(n-1)\bar{I}]$, where $I_{i}$ (i = 1,2, …,n) represents the recorded emission currents and the expression denotes the variance of emission current divided by the average value of the current ($\bar{I}$), were 0.30%, 0.31%, and 0.60%, respectively. It should be noted that the stepped instability in the current was only observed during the low current (Figure 4a,b) stabilization process, while the 50 nA stabilization process only exhibited current fluctuations. This can be attributed to the prolonged field emission and multiple thermal flashing treatments in the low current stages that had already removed most of the gas molecules existent at the tip. The duration of the stepped instability in the current also significantly decreased with the increase in the field emission current. The current value of up to 50 nA, combined with the application of high current treatments (sustained field emissions at over 100 nA for approximately 10 s) at the onset of this stage, readily facilitated energy transfer to the adsorbates and contaminants on the emitter surface under one single emission beam and a smaller emission area, assisting in their desorption from the surface and allowing for the gradual stabilization of the surface structure over the remaining time. Upon the completion of all stabilization processes, we observed stability at 50 nA with a fluctuation of 1.41%, and this was maintained for 2.5 h, as presented in Figure 4d.

The field emission stability of the ZrC nanoneedle shows clear advantages over the commercial W(310) filament in terms of emission current decay and fluctuation. For the W(310) emitters, even under an extremely high vacuum (EHV, 10^−9^ Pa), the emission current drops by 5% in 2.5 h, with current fluctuations increasing from 1.0% to 3.2% over time. In contrast, the ZrC nanoneedle exhibited no observable current decay at a vacuum of 10^−7^ Pa, which maintained a steady current fluctuation of around 1.5% during its prolonged emission. This performance is expected to improve further in higher vacuum conditions.

## 4. Conclusions

A ZrC nanoneedle field emitter was fabricated using an efficient and controllable SEM-FIB process, achieving a length exceeding 2 μm and a tip with a radius of curvature less than 20 nm. Under high-vacuum conditions of 10^−7^ Pa, the ZrC nanoneedle emitter exhibited a turn-on field of 6.5 V nm^−1^ with an emission current of 50 nA and achieved an emission current density as high as 1.4 × 10^10^ A m^−2^. The FEM pattern showed a single bright and well-focused emission electron beam. Additionally, the ZrC nanoneedle exhibited outstanding emission stability, fluctuating by only 1.41% after 150 min of continuous emission with a current of 50 nA in a 10^−7^ Pa vacuum. The stable emission is dependent on the interplay of a low work function, ideal nanoneedle morphology, and appropriate surface treatment. These results satisfy the requisite application standards for practical deployment in electron-beam devices.

## Data Availability

The original contributions presented in the study are included in the article, and further inquiries can be directed to the corresponding author/s.
